# The relevance of magnesium homeostasis in COVID-19

**DOI:** 10.1007/s00394-021-02704-y

**Published:** 2021-10-23

**Authors:** Valentina Trapani, Andrea Rosanoff, Shadi Baniasadi, Mario Barbagallo, Sara Castiglioni, Fernando Guerrero-Romero, Stefano Iotti, André Mazur, Oliver Micke, Guitti Pourdowlat, Giuliana Scarpati, Federica I. Wolf, Jeanette A. Maier

**Affiliations:** 1 Sezione di Patologia Generale, Dipartimento di Medicina e Chirurgia Traslazionale, Fondazione Policlinico Universitario A. Gemelli IRCCS-Università Cattolica del Sacro Cuore, Largo F. Vito 1, 00168 Rome, Italy; 2 Alleanza Contro Il Cancro, Rome, Italy; 3 CMER Center for Magnesium Education and Research, Pahoa, Hawaii USA; 4 Tracheal Diseases Research Center, National Research Institute of Tuberculosis and Lung Diseases (NRITLD), Shahid Beheshti University of Medical Sciences, Tehran, Islamic Republic of Iran; 5 Geriatric Unit, Department of Medicine, University of Palermo, Palermo, Italy; 6 Dipartimento di Scienze Biomediche e Cliniche L. Sacco, Università di Milano, Via G.B. Grassi 74, 20157 Milan, Italy; 7 Biomedical Research Unit of Mexican Social Security Institute, Durango, Mexico; 8 Department of Pharmacy and Biotechnology (FaBit) National Institute of Biostructures and Biosystems, Università di Bologna, Bologna, Italy; 9 Unité de Nutrition Humaine, INRAE, UNH, Université Clermont Auvergne, Clermont-Ferrand, France; 10 Department of Radiotherapy and Radiation Oncology, Franziskus Hospital, Bielefeld, Germany; 11 Chronic Respiratory Diseases Research Center, National Research Institute of Tuberculosis and Lung Diseases (NRITLD), Shahid Beheshti University of Medical Sciences, Tehran, Islamic Republic of Iran; 12 Anestesiologia e Rianimazione, Dipartimento di Medicina e Chirurgia, Università Degli Studi di Salerno, Fisciano, Italy

**Keywords:** Aging, Chronic non-communicable diseases, Immune response, Long COVID, Magnesemia, SARS-CoV-2

## Abstract

**Purpose:**

In less than one and a half year, the COVID-19 pandemic has nearly brought to a collapse our health care and economic systems. The scientific research community has concentrated all possible efforts to understand the pathogenesis of this complex disease, and several groups have recently emphasized recommendations for nutritional support in COVID-19 patients. In this scoping review, we aim at encouraging a deeper appreciation of magnesium in clinical nutrition, in view of the vital role of magnesium and the numerous links between the pathophysiology of SARS-CoV-2 infection and magnesium-dependent functions.

**Methods:**

By searching PubMed and Google Scholar from 1990 to date, we review existing evidence from experimental and clinical studies on the role of magnesium in chronic non-communicable diseases and infectious diseases, and we focus on recent reports of alterations of magnesium homeostasis in COVID-19 patients and their association with disease outcomes. Importantly, we conduct a census on ongoing clinical trials specifically dedicated to disclosing the role of magnesium in COVID-19.

**Results:**

Despite many methodological limitations, existing data seem to corroborate an association between deranged magnesium homeostasis and COVID-19, and call for further and better studies to explore the prophylactic or therapeutic potential of magnesium supplementation.

**Conclusion:**

We propose to reconsider the relevance of magnesium, frequently overlooked in clinical practice. Therefore, magnesemia should be monitored and, in case of imbalanced magnesium homeostasis, an appropriate nutritional regimen or supplementation might contribute to protect against SARS-CoV-2 infection, reduce severity of COVID-19 symptoms and facilitate the recovery after the acute phase.

## Introduction

As of June 18, 2021, more than 176 million cumulative confirmed COVID-19 cases have been reported, claiming more than 3.5 million lives all over the world [1], and the numbers are still growing. At an unprecedented speed, vaccines were developed and approved for immunization [2]. Although about 2,310,000,000 vaccines have been administered, we are far from the eradication of the disease. In addition, evidence is accumulating about the lingering effects experienced by recovered COVID-19 patients, even after a mild to moderate disease [3]. While scientists are searching for a specific therapy to treat the disease, there is an urgent need to individuate strategies to reinforce the immune system and prevent the infection, to mitigate the progression of the disease, and to ameliorate symptoms of long COVID. A balanced diet is essential to strengthen immune responses and to harmonize the microbiota, a complex ecosystem important for health [4]. The nutritional status of the patient influences the course of COVID-19, however knowledge about the nutritional support of COVID patients is very limited [5].

When the current SARS-CoV-2 pandemic began in early 2020, the global magnesium researcher group (MaGNet [6]) was alerted by the striking similarities between COVID-19 risk factors and conditions linked to magnesium deficit state in humans [7–9] such as age, diabetes, obesity, high blood pressure, arrhythmias, thrombosis and cardiovascular diseases. These conditions, which are associated with high odds of COVID-19 mortality [10, 11], are common in Western Societies and spreading globally. Indeed, food processing losses resulting in lower dietary magnesium intakes, reduced magnesium availability in the soil and, therefore, in the food chain, long-term prescribed drugs such as proton pump inhibitors, all might precipitate subclinical magnesium deficiency [12].

Moreover, we reasoned that magnesium deficiency might play a role in the pathophysiology of COVID-19. Even if the respiratory track is the primary site of the disease, the virus can disseminate to other tissues and complicate the clinical picture, eventually culminating in multi-organ failure [13]. Key mechanisms implicated in the disease include direct viral cytotoxicity, endothelial dysfunction and exaggerated release of inflammatory cytokines. Very early after the outbreak of the pandemic, it became evident that endotheliopathy is present in COVID-19 and is likely to be associated with critical illness and death, to the point that evaluation of endothelial markers is recommended and, in parallel, therapies are sought to preserve endothelial function [14, 15]. Notably, low magnesium induces a pro-inflammatory, pro-thrombotic phenotype in endothelial cells [16] and promotes platelet aggregation, release of beta-thromboglobulin and thromboxanes, all events that favor the development of thromboembolism [17]. Endothelial injury and hypercoagulable state are key components of the Virchow’s triad and might explain the high incidence of thromboembolic events in COVID-19 patients. Moreover, magnesium deficits may exacerbate the inflammatory response induced by SARS-CoV-2, maintaining and propagating the so-called cytokine storm, a reaction that leads to acute respiratory distress syndrome (ARDS), accentuates endothelial dysfunction and coagulopathy, and promotes multiple organ failure. In addition, other symptoms reported by COVID-19 patients, such as asthenia, myalgias, anxiety, depression, insomnia, might be attributable to magnesium deficiency [7].

PubMed and Google Scholar were used to pinpoint studies and articles on the topic of interest. Journal articles published between 1990 and June 2021 were reviewed. Keywords included “magnesium”, “Sars-CoV-2”, “COVID-19”, “long COVID”, “diabetes”, “obesity”, “aging”, “infectious diseases”, “hypertension”, “atherosclerosis”, “hypomagnesemia”, “thrombosis”. We selected only papers written in English and covering in vitro, animal and human studies.

On these bases, we discuss the physiological role of magnesium, the link between magnesium deficiency and chronic non-communicable diseases which represent a risk factor for severe COVID-19, the potential contribution of a low magnesium status to the pathogenesis of the disease to conclude with a rapid overview of clinical studies available in the literature and a summary of ongoing research on the topic.

### Magnesium, a metabolite

The role of magnesium in cell and tissue metabolism is complex and multifactorial. Accumulating evidence suggests that magnesium acts primarily as a key signaling element and metabolite in cell physiology. Consequently, the concept that magnesium is an electrolyte is too simplistic and obsolete [18–22]. Magnesium is involved in all metabolic and biochemical pathways and is required in a wide range of vital functions, such as bone formation, neuromuscular activity, signaling pathways, bioenergetics, glucose, lipid and protein metabolism, DNA and RNA stability, and cell proliferation. The enzymatic databases list more than 600 enzymes with magnesium indicated as cofactor, and additional 200 are reported in which magnesium acts as an activator [23]. However, it should be specified that, since it interacts directly with the substrate, magnesium is itself a substrate rather than a cofactor [20, 24]. Indeed, since Mg^2+^ binds to the phosphate moieties of metabolites, the phosphorylated molecules (i.e., ATP, phosphocreatine, as well as all the other phosphometabolites including those related to carbohydrate metabolism and cellular bioenergetics) form a complex with magnesium. This implies that the actual substrates of the biochemical reactions involving these metabolites are magnesium complexes. This is the reason why magnesium should be regarded as a metabolite and not as a cofactor acting in ancillary fashion in biochemical reactions [20]. As a paradigmatic example, the binding between ATP and Mg^2+^ results in an adequate conformation that allows to weaken the terminal O–P bond of ATP, thereby facilitating the transfer of phosphate [23]. Hence, the “active species” is not ATP, but MgATP^2−^ [20]. This concept is further sustained by the fact that, differently from other ions for which cells maintain transmembrane gradients, intra- and extracellular free magnesium (Mg^2+^) concentrations are comparable [24]. The total (bound + free) intracellular magnesium concentrations range from 10 to 30 mM. However, since most magnesium is bound to polynucleotides, ATP, phosphorylated metabolites and proteins, the concentration of its intracellular ionic (free) form falls in the range of 0.5–1.2 mM [25]. The little amount of intracellular [Mg^2+^], as compared to the intracellular [Na^+^] and [K^+^], which are in the order of 50 and 150 mM respectively [20], strengthens the evidence that the contribution of magnesium to the electric charge of the cell is almost negligible. Therefore, it is time to revise the concept that magnesium is an electrolyte.

The total body magnesium of an adult is approximately 25 g, of which 50–60% is in bone, and the remaining 40%–50% is in the soft tissues, with less than 1% present in the blood [25]. Magnesium is mostly absorbed by the small intestine and to lesser extent by the large intestine, and excreted by kidney through the finely tuned activity of many Mg^2+^ transporters [26].

Serum magnesium concentration in blood can be efficiently buffered by renal excretion and, in part, by its release from bones. In addition, magnesium is an intracellular cation whose content depends on the tissue and its metabolic state [27, 28]. Therefore, serum magnesium values within the reference range may not rule out a systemic magnesium-depleted state, and the clinical impact of magnesium deficiency may be easily underestimated [27–29]. Nevertheless, in the absence of a satisfactory and easily measurable biomarker to assess magnesium state, we have to rely on serum magnesium levels, which are found to be altered in many diseases.

Unfortunately, serum magnesium levels are still not determined routinely in daily clinical practice. Worldwide, they are measured primarily in critically ill subjects and sometimes in elderly people at hospital admission. In view of the large variety of pathophysiological conditions associated to a decrease of magnesium availability, we strongly suggest to include magnesium in the evaluation of the blood ionogram.

### Magnesium deficit in aging and non-communicable chronic diseases

The overall prevalence of hypomagnesemia in geriatric patients reaches 36% [30], among diabetic patients ranges from 19% to 29% [31, 32], and in patients with high blood pressure, it has been found to be 63.8% [33]. In addition, it has been reported that magnesium deficiency is associated with obesity and the increased incidence of hypertension, cardiovascular disease, congestive heart failure, arrhythmias, and chronic kidney disease, particularly in patients under dialysis [34–36]. Furthermore, it has been noted that mortality by cardiovascular deaths in subjects with hypomagnesemia is significantly higher as compared with individuals exhibiting normal magnesium levels [37]. Within this context, hypomagnesemia, at the time of admission in the Intensive Care Unit (ICU), is associated with the length of stay, need for mechanical ventilation, and mortality [38].

Because patients with diabetes, particularly the elderly with type 2 diabetes (T2DM), are at a higher risk of severe disease or death due to SARS-CoV-2 infection than age-matched individuals without diabetes, it is of interest to recall that a consistent body of evidence has linked magnesium deficiency to alterations of insulin sensitivity, including metabolic syndrome and T2DM [39]. Indeed, T2DM has been associated with several magnesium abnormalities [40, 41]. An umbrella review to map and grade health outcomes linking to magnesium intake and supplementation confirmed that an elevated magnesium intake is associated with a decreased risk of T2DM [42].

Finally, it is important to highlight that magnesium modulates the contractile state of bronchial smooth muscle cells. Hypomagnesemia increases airway reactivity susceptibility to recurrent upper respiratory tract infections, and development of pulmonary fibrosis [7, 43]. Magnesium depletion triggers bronchial contraction and spasm, while magnesium supplementation produces bronchial relaxation [40, 44]. Several possible mechanisms have been postulated for the positive magnesium action to relax bronchial smooth muscle, such as its calcium channel blocking action, a decreased sensibility to the depolarizing action of acetylcholine, a stabilization of mast cells and T lymphocytes, and a stimulation of nitric oxide and prostacyclin release [40]. Accordingly, nebulized magnesium sulfate has been used as a bronchodilator agent in adjuvant care of asthmatic patients [45]. Moreover, because of its vasodilatory, anti-inflammatory and anti-thrombotic effects, magnesium may reduce the respiratory symptoms and improve lung function of COVID-19 patients. All these effects might contribute to the reduction of the ventilation-perfusion mismatch, which is one of the most important reasons for hypoxemia in COVID-19 patients, and to the improvement of oxygenation in these patients [46]. Additionally, because of the emerging role of mastocytes in driving diffuse alveolar injury in COVID-19 [47], it should be recalled that magnesium reduces mastocyte degranulation and, subsequently, prevents the release of inflammatory, pro-thrombotic and fibrotic mediators [48]. Accordingly, famotidine, an antagonist of histamine H2 receptors, seems to mitigate COVID-19 symptoms caused by dysfunctional mast cell activation and histamine release [49].

A recent paper demonstrates that low magnesium reduces diaphragm function in an experimental model of sepsis [50]. If confirmed in humans, these results suggest that magnesium deficiency might lead to the need of assisted ventilation by impairing diaphragm contraction and, therefore, breathing.

On these bases, there is a plausible pathophysiological basis to support that magnesium is among the missing keys linking chronic diseases with the severity and prognosis of COVID-19.

### Magnesium in infectious diseases

Most findings regarding the direct association between magnesium status and immune function are derived from animal models. In humans, the fundamental importance of magnesium in immunity and the underlying mechanisms have been highlighted by the discovery of the primary immunodeficiency XMEN (X-linked immunodeficiency with magnesium defect, Epstein–Barr virus infection, and neoplasia), which is characterized by chronic Epstein-Barr virus infection [51]. However, general evidence that magnesium deficiency may confer a higher predisposition to infectious diseases is mostly indirect [34]. An optimal magnesium status is necessary for the synthesis, transport, and activation of vitamin D [52]. Vitamin D is an immunomodulatory hormone, and vitamin D deficit has been linked to various infectious diseases, including upper respiratory and enteric infections, pneumonia, otitis media, Clostridium infections, vaginosis, urinary tract infections, sepsis, influenza, dengue, hepatitis B, hepatitis C, and HIV infections [53]. Accordingly, a number of clinical trials aiming to determine the efficacy of administration of vitamin D and its metabolites for treatment of these diseases have been conducted, but have rarely shown benefits as therapeutic or preventive measures. Nevertheless, an important exception to the general trend is for upper respiratory tract infections. A meta-analysis involving 25 randomized controlled trials (RCTs) and data from 10,933 participants showed that vitamin D supplementation reduced the risk of acute respiratory tract infection among all participants [54]. The close relationship between magnesium and vitamin D suggests that the higher incidence of infectious diseases associated with vitamin D deficiency can be at least in part explained by a deficit of magnesium, and this may be particularly relevant in the respiratory tract.

Altered magnesium status also seems to have a prognostic role in older people affected by bacterial pneumonia. Hypomagnesemia and hypermagnesemia were both associated with excessive short-term mortality, 18.4% and 50%, respectively, compared to normal values of serum magnesium [55]. Moreover, low serum magnesium status was a significant predictor of frequent readmissions for acute exacerbation of chronic obstructive pulmonary disease in a retrospective study on older adults [56].

More relevantly, in a cohort of 144 adults who were hospitalized with SARS-CoV-1 infection in the greater Toronto area in the 2003 epidemic, hypomagnesemia was found in many patients on admission, and tended to worsen during hospitalization [57]. It was unclear whether this represented the natural history of the disease or was secondary to effects of ribavirin or other therapies on renal tubular function. Indeed, after adjusting for SARS-CoV-1 ‐related prognostic factors and corticosteroid use, ribavirin use in 306 SARS patients was strongly associated with hypomagnesemia, and the risk of hypomagnesemia attributable to ribavirin use was 45% [58]. Since ribavirin is also currently employed for compassionate use in COVID-19 patients with respiratory distress, these findings stress the importance of evaluating and, if necessary, correcting magnesemia. Although in a different context, an animal study suggested that hypomagnesemia might be a common adverse effect of other antiviral therapies, and might potentiate the risk of cardiovascular complications, while magnesium supplementation is protective [59].

On the other hand, in quest of more effective first-line treatments for COVID-19, several in silico docking studies have been published for the repurposing of existing antivirals. Interestingly, binding of several candidate drugs to their viral target is dependent on magnesium, which is not surprising in view of its biochemical properties [60, 61]. It is tempting to speculate that the pharmacological activity of the drugs under investigation for treatment of COVID-19 patients might be dependent on the intracellular magnesium concentration and that magnesium deficits may reduce efficacy.

In conclusion, very little is known on the prevalence and significance of hypomagnesemia in infectious diseases. Nevertheless, the current pandemic has raised unprecedented research efforts that are unveiling intriguing relationships between magnesium status and the clinical course of COVID-19.

### Links between magnesium derangements and COVID-19 pathogenesis

**Fig. 1:**
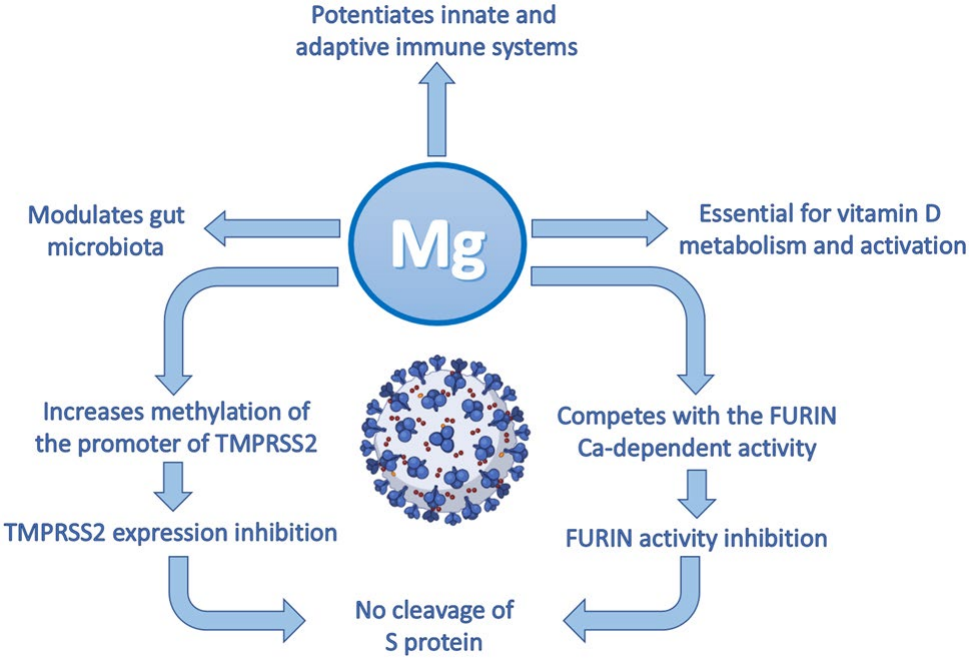
How magnesium might contribute to protecting against COVID-19. Magnesium modulates innate and adaptive immunity and gut microbiota composition, and is essential for Vitamin D activation and its consequent anti-inflammatory activity. Crucial steps of SARS-CoV-2 infection might be directly affected by magnesium

As knowledge about the pathogenesis of COVID-19 has increased, more hints illuminate the potential role of dysregulated magnesium homeostasis in the prevention and clinical trend of the disease (Fig. 1).

First issue to recall is that magnesium tunes the activity of the innate and adaptive immune systems.

It stabilizes the membranes of mastocytes, localized in the submucosa of the airways and the alveolar septa, thereby preventing their degranulation and the release of a myriad of mediators [62]. Magnesium regulates the activity of neutrophils and macrophages, inhibits their priming and oxidative burst, and also hinders the Toll-like receptor 4/NFκB axis [62]. In lymphocytes, magnesium controls the amounts of IP3 and DAG, important second messengers activated after the engagement of B and T cell receptors [63]. Importantly, magnesium protects against viral infections because adequate intracellular magnesium concentrations are required for the cytotoxic activity of T lymphocytes and natural killer (NK) cells [64]. Beyond potentiating and harmonizing immune function, it is emerging that magnesium levels might influence initial events of SARS-CoV-2 infection. The viral spike (S) protein binds the entry receptor, the angiotensin-converting enzyme 2 (ACE2), expressed in many tissues, thus accounting for pulmonary and extrapulmonary manifestations of COVID-19 [13]. For fusion between viral and cell membranes to occur, the S protein needs to be proteolytically cleaved by host cell proteases, mainly the transmembrane protease serine protease 2 (TMPRSS2) and the pre-protein convertase furin [65]. Magnesium might play a role in inhibiting the activity of these enzymes. Preliminary results suggest that magnesium treatment increases the methylation of the promoter of TMPRSS2, thus hindering transcription and, consequently, reducing the expression of the enzyme [66]. Moreover, thanks to its calcium antagonistic function, magnesium might hamper the activity of the calcium-dependent furin [67]. It follows that magnesium deficiency may promote virus infectivity. Once the infection is ongoing, magnesium deficiency can exacerbate the inflammatory response contributing to the so-called cytokine storm that is involved in the pathogenesis of severe SARS-CoV-2 clinical manifestations. The immune system reaction to the coronavirus may be so overwhelming and inappropriate as to jeopardize the lungs and the other tissues more than the virus itself, endangering the survival of patients. Notably, low levels of magnesium associate also with increased amounts of inflammatory mediators [16, 62], among which platelet activating factor (PAF) [68]. PAF is a key player in COVID-19, being implicated in generating microthrombosis and promoting respiratory distress [69, 70].

Although some controversies exist, a recent metanalysis reports that low serum levels of 25-hydroxyvitamin D (25-OHD) are associated with a higher susceptibility to SARS-CoV-2 infection, COVID-19 severity and mortality [71]. Indeed, as highlighted in the previous section, vitamin D is necessary for a healthy immune system. The activity of vitamin D is strictly intertwined with magnesium [34]. Clearly, magnesium deficiency leads to reduced amounts of active vitamin D, thus generating a fertile soil for SARS-CoV-2 infection.

A last issue to mention is the recent finding that magnesium deficiency alters the delicate balance between gut microbiota and the host [72, 73], thus influencing the airway microbiota and the immune response [74]. Lately, the relation between microbiota dysbiosis and COVID-19 severity has attracted the attention of the scientific community [75, 76] and hopefully new hints will soon be available.

### Magnesium in COVID-19: clinical studies

As delineated in the previous sections, the key role of magnesium in maintaining proper immune, vascular and pulmonary function strongly supports the hypothesis that magnesium status may affect the susceptibility and the response to SARS-CoV-2. Indeed, a few recent reports have analyzed magnesium levels in COVID-19 patients and assessed their association with disease outcomes (Table 1).

**Table 1:** Existing clinical studies showing alterations of magnesium levels in COVID-19 patients

Study Design	Number of patients	Outcome	References
Retrospective	300	Severe cases have lower magnesium levels than mild and moderate cases	[73]
Retrospective	459	Low magnesium levels are associated to mortality	[74]
Prospective	300	48% of hospitalized patients are hypomagnesemic, but hypermagnesemia is more prevalent in ICU cases	[75]
Prospective	200	In pregnant women, SARS-CoV2 infection was associated to higher magnesemia in the first and third semester in comparison to non-infected subjects	[76]
Cross-sectional	58	An altered magnesium status (either hypomagnesemia or hypermagnesemia) was more frequent in ICU cases	[77]
Retrospective	90	Patients show levels of calcium and magnesium lower than controls	[78]
Retrospective	320	Serum calcium and magnesium concentration were significantly lower in hypokalemic than normokalemic patients	[79]
Case report	1	Persistent hypokalemia and hypomagnesemia, which continued for more than 5 months after the initial infection and its resolution	[80]

Zeng et al. [77] analyzed over 300 patients and found that severe cases (defined as those having oxygen saturation ≤ 93% at rest, or PaO_2_/FiO_2_ ≤ 300 mmHg, or requiring ICU care) showed significant lower levels of magnesium than mild and moderate cases, though all values remained within the reference range. Such difference was found consistently over the clinical course since disease onset. In addition, low magnesium levels were indicated as risk factor for mortality in COVID-19 patients. A retrospective research on a total of 459 confirmed cases found that in the 63 expired individuals magnesium levels on admission were significantly lower than in the 396 survivors (1.61 ± 0.19 vs. 1.81 ± 0.23 mg/dl, respectively) [78]. Quilliot et al. [79] performed a detailed analysis of magnesemia and associated factors in a cohort of 300 patients. Among these, 48% had a magnesemia below 0.75 mmol/L, including 13% of severely hypomagnesemic patients (0.65 mmol/L)^1^; on the other hand, a small proportion of patients (9.6%) was hypermagnesemic (> 0.95 mmol/L). In this study, in moderate cases of COVID-19, serum magnesium concentrations were significantly lower and the prevalence of hypomagnesemia was significantly higher than in critical cases, whereas the prevalence of hypermagnesemia was significantly increased in ICU cases. It is intriguing that in pregnant women, SARS-CoV2 infection was associated to significantly higher magnesemia in the first and third semester in comparison to non-infected subjects; however, magnesium levels did not correlate with disease severity in this cohort [80].

A frequent confounding factor in the cited studies may be that individuals with comorbidities, such as hypertension, cardiovascular diseases, diabetes, and obesity, who are more prone to develop severe COVID-19, often show altered magnesium levels. In this respect, it is worth mentioning a small cohort study by Sarvazad et al. [81]. They analyzed the electrolyte status in COVID-19 patients on hospital admission, but patients with a history of cancer, diabetes, hypertension, or cardiovascular and renal disorders were excluded from the study. Overall, out of 50 patients with no previous underlying disease, 32% were found to be hypomagnesemic (1.26%–1.7 mg/dl), 6% were severely hypomagnesemic (< 1.25 mg/dl) and 14% were hypermagnesemic (> 2.6 mg/dl). Most importantly, an altered magnesium status (either hypomagnesemia or hypermagnesemia) was more frequently found in the ICU cases compared to the outpatients.

Derangement in magnesium homeostasis may have multifold consequences. Secondary to hypomagnesemia, disturbances of potassium and calcium handling are often detected; when serum magnesium levels are low, hypokalemia and hypocalcemia may be refractory to treatment by supplementation unless magnesemia is concomitantly corrected [25]. Both K^+^ and Ca^2+^ imbalances have been described in COVID-19 patients, and in some instances were associated to altered magnesium levels. Al-Hakeim et al. [82] reported preliminary findings showing lower levels of both calcium and magnesium in 60 COVID-19 patients in comparison to 30 matched controls. Importantly, calcium and magnesium levels positively correlated with oxygen saturation levels. A retrospective, observational study was conducted on 320 non-critically ill patients [83]. In this cohort, serum calcium and magnesium concentration were significantly lower in hypokalemic than normokalemic patients, and 18.4% of hypokalemic patients was also frankly hypomagnesemic. Furthermore, patients who experienced hypokalemia had significantly longer hospitalization than controls. A case report [84] suggested that electrolyte imbalances may persist in the long term. A COVID-19 patient was admitted with low potassium levels, but normal magnesiuim levels, but during his hospitalization developed persistent hypokalemia and hypomagnesemia, which continued for more than 5 months after the initial infection and its resolution.

It is hard to extrapolate a univocal conclusion from the existing evidence, that was obtained from a variety of populations, employing different measuring techniques and different reference values. The long-standing debate about the magnesium reference range, as well as the lack of consensus about the most appropriate indicators of magnesium status, are just one side of the story [6]. The main problem lies in the paucity of studies that have been appositely designed to investigate the role of magnesium in COVID-19 clinical course. All the studies that we have reviewed so far have inherent flaws, mostly due to their observational nature. In most studies, anomalies in magnesium were simply incidental findings that did not undergo further analysis. Magnesium levels in adequately matched controls were rarely provided. Notably, in most cases, magnesium values were simply “snapshot” values, taken on admission or at an undetermined time during hospitalization. We do not know magnesium levels before infection, nor their evolution during the course of the disease. Information on nutritional habits, medications or dietary supplements is missing.

Despite all limitations, existing data seem to corroborate an association between deranged magnesium homeostasis and COVID-19. The occurrence of hypermagnesemia in ICU cases in the absence of nephropathy, evidenced by two studies [79, 81], is an intriguing finding that might be related to rapid mobilization from soft tissues in patients with sepsis or to necrotic events due to microvascular thrombosis. Therefore, this finding may not necessarily be in contrast with data that indicate hypomagnesemia as a risk factor for developing complications.

Unfortunately, at present we do not possess enough knowledge to answer to some fundamental questions. Is magnesium deficiency a risk factor for developing COVID-19? In other words, are magnesium-deficient individuals more prone to infection? Or vice versa, does infection lower magnesium levels? And in either case, do magnesium levels affect the clinical course of the disease? An affirmative and conclusive answer to these questions would offer new prophylactic or therapeutical opportunities by an intervention as simple as magnesium supplementation. Therefore, we urgently need to design further and better studies to clarify these issues. At present, we are aware of just one interventional study involving magnesium supplementation in COVID-19 patients that has been published [85]. In a cohort of 43 patients (> 50 years of age), seventeen patients received a combination of vitamin D, magnesium and vitamin B12 (DMB) before requiring any form of oxygen therapy, intensive care support, or both. The intervention group showed a significant reduction in the proportion of patients clinically deteriorating. The protective effect of DMB supplementation was retained after adjusting for age and hypertension. Further promising work is ongoing [86–88] (Table 2).

**Table 2:** Ongoing clinical studies investigating the role of magnesium in COVID-19

Study Design	Clinical Center	Number of Patients	Outcome
Interventional RCT [84]	Masih Daneshvari Hospital, Shahid Dr. Labbafinejad Hospital, Shahid Sadoughi Hospital, Iran	100	Improvement of respiratory function and symptoms upon inhalation of MgSO_4_
Interventional RCT	Mexican Social Security Institute at Durango, Mexico	100	Reduction of the probability of infection and the need for hospitalization and/or mortality upon administration of MgCl_2_ plus vitamin D
Interventional	University of Minnesota, USA	NA	Efficacy of Mg supplementation for preventing sudden cardiac death in hypertensive patients
Prospective	AOU San Giovanni di Dio e Ruggi D'Aragona, University of Salerno, Italy	100	Correlation between total and ionized serum Mg levels and outcomes in ICU patients
Prospective	National Research Institute of Tuberculosis and Lung Diseases, Iran	200	Correlation between serum Mg/Ca level and CRP in ICU patients
Retrospective and Prospective [85]	Academy of Nutrition and Dietetics / George Mason University, USA	250	Correlation between nutrition standard care practices and outcomes in ICU patients
Retrospective	University of Milan – Ospedale Sacco, Italy	430	Correlation between Mg levels and the course of COVID-19
Retrospective	AUSL-IRCCS Reggio Emilia, Italy	241	Correlation between Mg levels and COVID-19 outcomes
Retrospective [86]	University Hospital of Puebla, Mexico	118	Correlation between renal function, serum magnesium levels and mortality in T2D patients
Retrospective	Comenius University in Bratislava –Biomedical Centre Martin at Jessenius Faculty of Medicine, and the Hospitals of Žilina self-governing region, Slovakia	300	Correlation between Mg levels (and of other bio-elements) and the course of COVID-19
Retrospective	Indiana University, USA	NA	Associations between nutritional status of vitamin D, iron, and magnesium and disease severity of COVID-19
Cross-sectional	Mexican Social Security Institute at Durango, Specialty Hospital from the National Medical Center Century XXI, Mexican Social Security Institute at Mexico City, Mexico	450	Correlation between hypomagnesemia and COVID-19 mortality

*NA* Not available

### Magnesium and Post-Acute COVID-19 Syndrome

**Fig. 2:**
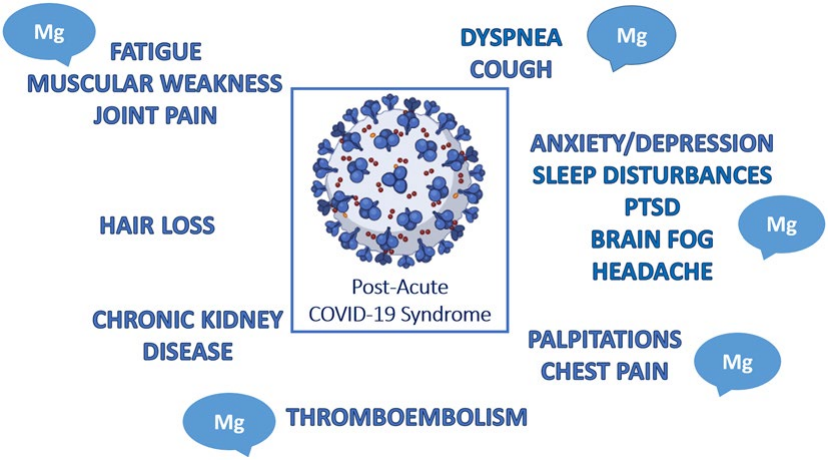
How magnesium deficiency might contribute to the long COVID-19 syndrome. Among the symptoms of post-acute COVID-19 syndrome summarized in the figure, many are often associated to hypomagnesaemia, i.e., muscular-related symptoms, pulmonary distress, neurologic and behavioral disorders, some arrhytmias, hypercoagulation disorders. These symptoms may benefit from a magnesium-rich diet or, if necessary, magnesium supplementation. *PTSD* Post Traumatic Stress Disorders, including physical, cognitive, and psychiatric symptoms

Similarly to post-acute viral syndromes observed in survivors of other virulent coronavirus epidemics (e.g., SARS and MERS), increasing reports describe persistent and prolonged effects after acute COVID-19 [3]. Long COVID affects the lungs, the brain, the heart, the gastrointestinal system, and the kidney. Patients most commonly experience hair loss, fatigue, muscular weakness and joint pain, arthralgia, followed by dyspnea/cough, and chest pain/palpitation. Neurological symptoms are also frequent: headache, sleep disorders, anxiety/depression and cognitive disturbances, in particular clouding of consciousness called “brain fog” (Fig. 2). According to epidemiological studies, a high percentage of hospitalized and non-hospitalized COVID-19 survivors have at least one symptom up to 2–6 months after infection. Overall, these symptoms lead to a decline in the quality of life and increase frailty, and represent a current concern calling for multidisciplinary management of the post-acute phase of the disease.

The mechanisms involved in the pathophysiology of long COVID syndrome are multifactorial and include direct virus-induced alterations, immunologic aberration and inflammatory damage in response to the acute infection, as well as ischemia due to microvascular thromboembolism, immobility and metabolic derangements, the latter being associated with the PTSD (Post Traumatic Stress Disorder). The simultaneous occurrence of chronic fatigue, dyspnea, pain and cough suggest the possibility of alterations in the brain. Interestingly, SARS-CoV-2 has neuro-invasion capacity and sensory neurons seem to be its entry point to the central nervous system (CNS) [89]. Furthermore, neuroinflammatory events driven by the cytokine storm are likely to affect various regions of the CNS [90].

In this context, it is noteworthy that in the brain magnesium affects multiple biochemical processes involved in cognitive functions, cell membrane stability and integrity, NMDA-receptor response to excitatory stimuli. It also exerts a calcium-antagonist action and combats neuroinflammation [40]. Consistently, magnesium deficit determines anxiety, insomnia, hyperemotionality, depression, headache, light-headedness [40], symptoms included in the post-acute COVID-19 syndrome. Moreover, magnesium deficits have been suggested to cause weakness and muscle pain. This is not surprising since magnesium is key for all the enzymes utilizing or synthesizing muscle ATP, and thus for the production of muscle energy, and also regulates contraction and relaxation [25]. Additionally, magnesium assures the regenerative capacity of skeletal muscle fibers [91].

In conclusion, an altered magnesium homeostasis might reasonably contribute to and aggravate long COVID syndrome (Fig. 2). Therefore, assessing and, if necessary, correcting magnesaemia is essential to support full recovery. It is clear that care for patients with COVID-19 does not end at the time of hospital discharge, and comprehensive care of recovered patients is needed in the outpatient setting as well.

In this respect, magnesium supplementation is a safe and cost-effective intervention that could help restoring the severely deranged homeostatic equilibrium of the body.

## Conclusions

The ongoing SARS-CoV-2 pandemic dramatically impacted on health and economy, and has significantly constrained social and cultural rights. Intervention strategies to cope with SARS-CoV-2 infection are in their early stages and we need proof of clinical efficacy. We propose to consider the relevance of magnesium, frequently overlooked in clinical practice. It is therefore relevant to include magnesium in the ionogram to diagnose alterations of magnesium homeostasis, which might represent one of the many dowels that contribute to complete the complex mosaic of signs and symptoms of acute and long COVID-19.

Monitoring and restoring magnesium homeostasis through an appropriate nutritional regimen or eventually by supplementation should therefore be taken in account for the general population, in particular during the current pandemic, as magnesium might contribute to prevent SARS-CoV-2 infection, to reduce severity of COVID-19 symptoms and to facilitate the recovery after the acute phase.

## Footnotes

1 Magnesemia is expressed in mmol/L or mg/dL (conversion: mmol/L = mg/dL × 0.4114).
